# Impact of Organic and Conventional Vegetable‐Based Curing Ingredients on Frankfurter Quality and Sensory Attributes

**DOI:** 10.1002/fsn3.70148

**Published:** 2025-04-04

**Authors:** Siyuan Sheng, Steven C. Ricke, Erin M. Silva, James R. Claus

**Affiliations:** ^1^ Department of Animal & Dairy Sciences University of Wisconsin‐Madison Madison Wisconsin USA; ^2^ Department of Plant Pathology University of Wisconsin‐Madison Madison Wisconsin USA

**Keywords:** alternative curing, frankfurter, GC–MS/MS, HPLC, nitrite, organic

## Abstract

Frankfurters, a widely consumed cured meat product in the United States, provide an ideal model for assessing the effects of conventional and organic plant‐based curing ingredients. This study aimed to evaluate the impact of commercially available conventional and organic plant‐based curing ingredients on the quality and sensory characteristics of frankfurters. Five nitrite source treatments (TRT)s were analyzed: preconverted celery (CEL), organic celery (OCEL), Swiss chard (SW), organic Swiss chard (OSW), and sodium nitrite (SN). Consumer sensory panel scores revealed a subtle variation in the perception of non‐meat aftertaste among alternative cured frankfurter TRTs. No difference (*p* > 0.05) was observed in overall liking and purchase intent among all TRTs. Furthermore, the consumer sensory panel indicated that OSW had a marginally higher (*p* = 0.077) non‐meat aftertaste compared to SW. Analysis of volatile compounds offered detailed insights into the interactions and effects of sodium nitrite and plant‐based curing powders on finished products. Esters and terpenoids were strongly positively correlated (*r* > 0.75) with non‐meat aftertaste, whereas alcohols, amino acids, and aldehydes were strongly negatively correlated (*r* < −0.75) with non‐meat aftertaste. This study found that some commercially available organic curing ingredients may go through a deodorization process, resulting in an undetectable non‐meat aftertaste. The combined findings indicated that even though non‐meat aftertaste is discernible in organic versus conventional cured meat products, it does not affect consumers' overall liking or purchase intent for vegetable‐based curing ingredients.

## Graphical abstract

No significant differences in overall liking and purchase intent from consumers regarding conventional and organic cured frankfurters. Curing ingredients affect sensory aroma and non‐meat aftertaste. Distinct VOC profiles among all treatments.

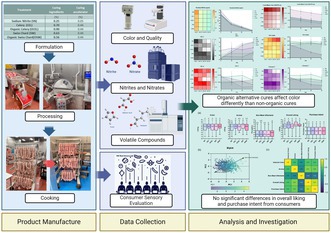

## 1 Introduction

The term “frankfurter” traces back to Frankfurt, Germany, where these sausages were first made in the 13th century. German immigrants introduced frankfurters to the United States in the 19th century, where they evolved into various formulations incorporating pork, beef, and poultry (Kraig 2009). The traditional German frankfurter is a thin sausage made from pure pork, with a characteristic flavor profile achieved through specific spices and various smoking techniques (Allen 2015). In the United States (USA), the recipe was adapted to include beef, pork, poultry, and a mixture of these meats, reflecting the diverse palate of U.S. consumers (Miller et al. 2021; Willits‐Smith et al. 2023). The inclusion of these additional meats and various seasonings led to a wide variety of hot dog styles across different regions. Traditional curing methods use synthetic sodium nitrite and sodium erythorbate to preserve meat and enhance flavor (Rasmussen and Sullivan 2019). These compounds have been well‐documented for their efficacy in preserving meat from lipid oxidation (Karwowska et al. 2019), providing characteristic cured color and flavor (Froehlich et al. 1983), and preventing the growth of pathogenic bacteria, including *Clostridium botulinum* and other harmful microorganisms (Lebrun et al. 2020).

The organic meat market has reached an estimated size of $1.5 billion. Individuals who consumed organic or natural products before the COVID‐19 pandemic have maintained or increased their consumption postpandemic (Brata et al. 2022; The Organic Center 2019). Consequently, the growing demand for natural foods has led to the development of alternative curing methods using plant‐based nitrate sources, such as celery juice or powder (Arsenault 2019). Celery powder has been used in the organic meat industry as an alternative meat curing ingredient since the inception of the USDA National Organic Program (U.S. Department of Agriculture 2024). These organic curing agents are derived from vegetable sources and are perceived as healthier options by consumers due to their natural origin and absence of synthetic additives. The National Organic Standards Board (NOSB) currently permits the use of nonorganic celery powder in organic products. However, the NOSB is in the process of conducting sunset reviews to transition to organic sources of celery powder. This involves evaluating whether nonorganic celery powder should remain on the National List of Allowed and Prohibited Substances or be replaced with organic alternatives (National Organic Standards Board 2023).

Recent studies have highlighted the impact of different natural source curing agents on the sensory attributes and consumer acceptance of processed meats (Jin et al. 2018; Sullivan et al. 2012). Alternative curing using plant‐derived nitrite sources can influence the final product's flavor profile and non‐meat aftertaste due to the presence of plant phytochemicals and their fermented derivatives (Siekmann et al. 2021). The chlorophyll and carotenoid content in organically grown vegetables can vary depending on cultivation practices (Chausali and Saxena 2021; Yu et al. 2018), potentially affecting the color of the frankfurters. It is unknown if the presence of pigments, phenolics, and polyphenols in vegetable‐based curing powders could affect the flavor of alternative cured frankfurters. Moreover, it remains unclear whether the development of volatile organic compounds (VOCs) during cooking could further influence consumer perceptions and preferences. By investigating the influence of both conventional and organic curing methods, this research aims to provide insights into how these practices affect the sensory properties and overall consumer acceptance of frankfurters. This study will contribute to the understanding of conventional and organic meat curing ingredients that cater to the evolving market trends and consumer preferences for organic meat products and further contribute to the development of the organic meat market. Thus, the objective of this study was to investigate the effects of conventional and organic curing agents on the quality and sensory attributes of frankfurters. This includes physicochemical analysis (proximate analysis, color, and cure efficiency), sensory evaluation of consumer preferences, and analysis of volatile organic compounds (VOCs). Understanding these differences is crucial for regulators and meat processors aiming to meet consumer demands for both traditional and organic products while ensuring excellence in product quality.

## 2 Material and Methods

### 2.1 Experiment Design

A total of five treatments (TRT)s that consisted of plant source nitrates and a sodium nitrite (SN) control were incorporated into the experimental design. An ingoing nitrite concentration of 156 ppm of equivalent nitrite was formulated to a 11.34 kg meat block coming from a synthetic chemical curing ingredient (Sure Cure, comprised of salt, 6.25% SN, FD&C Red #3; Excalibur Seasoning, Wichita, KS, USA), and vegetable source curing powders include both conventionally grown celery (CEL) and Swiss chard (SW) as well as organically grown celery (OEL) and Swiss chard (OSW). TRTs were formulated with 156 ppm of sodium nitrite or equivalent concentration for alternative cure TRTs. Meat curing was accelerated with sodium erythorbate (547 ppm) for sodium nitrite (SN) TRT and cherry powder for vegetable source nitrite TRTs.

Physicochemical properties' data including cured meat pigments (CMP), total meat pigments (TMP), total myoglobin content (TMC), and proximate analysis data (moisture, fat, and protein) were analyzed on Day 0 with duplicate measurements. Objective color, residual nitrite, and pH were assessed on a 15‐day interval from Day 0 to Day 45 with triplicate measurements to understand the trend of quality changes during storage. An IRB‐approved sensory evaluation was conducted between Days 14 and 21.

### 2.2 Product Formulation and Manufacturing

Frankfurters were formulated to contain 156 ppm sodium nitrite or an equivalent amount when cured with vegetable nitrite source curing powder. Nitrite concentration was measured for all commercially available vegetable nitrite source curing powders prior to formulation. The concentration of nitrite ion equivalent was determined to be 21487.1, 18436.1, 23145.3, and 21335.6 ppm for CEL, SW, OCEL, and OSW, respectively. The curing accelerator was added at 547 ppm sodium erythorbate for the SN and 547 ppm ascorbate equivalent for the treatments (TRTs). Frankfurters were manufactured with 85% lean fresh beef trimming and 50% lean pork trimming, sourced from a local supplier with a recent pack date. All treatments (TRTs) were formulated accordingly (Table 1) and manufactured in random order.

**TABLE 1:** Treatment and control formulations for frankfurters.

Treatment^a^	Water (%)	Salt (%)	Dextrose (%)	Sodium tripolyphosphate (%)	Spice mix (%)	Curing ingredients (%)	Curing accelerator (%)	Total NMI (%)
SN	19.20	1.73	0.77	0.38	0.47	0.25	0.05	22.85
CEL	18.36	1.73	0.77	0.38	0.47	0.70	0.44	22.85
OCEL	18.16	1.73	0.77	0.38	0.47	0.90	0.44	22.85
SW	18.43	1.73	0.77	0.38	0.47	0.63	0.44	22.85
OSW	18.50	1.73	0.77	0.38	0.47	0.56	0.44	22.85

*Note:* Nonmeat ingredients (NMI) were mixed with 11.34 kg of meat ingredients (5.67 kg of 50% lean fresh pork trim and 5.67 kg of 85% lean fresh beef trim). NMI percentages were relative to the total weight of meat ingredients.

Abbreviations: CEL, celery powder; OCEL, organic celery powder; OSW, organic Swiss chard powder; SN, sodium nitrite; SW, Swiss chard powder.

^a^ Treatment abbreviations refer to nitrite sources.

All meat (beef and pork) trimmings were first mixed in a meat mixer, followed by transferring to a bowl chopper. After chopping, the emulsion for each TRT was stored overnight in a temperature‐controlled cooler (34°C), covered with moisture‐impermeable wax‐coated butcher paper. The emulsion was subsequently transferred to a rotary vane vacuum‐filling machine (Model VF616 Vacuum Stuffer, Handtmann Inc., Lake Forest, IL., USA) with an automatic linking attachment and stuffed into 33 mm moisture‐impermeable plastic casings to prevent chemical cross‐contamination during thermal processing. Cooking was conducted using a common frankfurter smokehouse schedule, reaching an internal temperature of 71.2°C or above. After thermal processing, the TRTs were chilled in a cooler (2°C). Frankfurters were then placed in 4‐mil thick barrier bags (Uline S‐19920 Vacuum Bag, Uline Inc., Pleasant Prairie, WI., USA) and vacuum packaged at full strength for 25 s (Multivac C 500 Double chamber machine, The Multivac Group Kansas City, MO, USA) for a 60‐day sampling period at 2°C prior to all sensory and physicochemical analyses.

### 2.3 Proximate Composition

Proximate composition was measured on samples from all treatments which included crude protein, fat, and moisture using Association of Official Analytical Chemists (AOAC) procedures. Fat and moisture content in samples were analyzed using a meat analyzer (CEM Smart 6 Meat Analyzer, CEM Co., Matthews, NC, USA) with an automatic calibration function (AOAC Official Method 2008.06). Protein content in the sample was analyzed using a nitrogen analyzer (Leco 828 series, LECO Corporation, St. Joseph, MI, USA) using Kjeldahl methods (AOAC 981.10). Samples for proximate composition analysis were collected on Day 0 for all measurements and kept frozen (−80°C). Samples were prepared in duplicate and measured on frozen ground samples from Day 0 for all measurements. The amount of added water in finished products was calculated according to the USDA method (U.S. Department of Agriculture 1990) for determining added water in meat products, as outlined in 9 CFR 318.22. This calculation is based on the principle that lean muscle has a natural moisture‐to‐protein ratio of approximately 4:1. The formula used is: % moisture minus (four times % protein).

### 2.4 Objective Color Measurement

Color measurements were taken using a handheld vertical portable spectrophotometer (Konica Minolta CM‐600d, Konica Minolta Inc., Chiyoda, Tokyo, Japan) with a 2° standard observer. The color was measured using the Commission Internationale de I'Eclairage (CIE) L* (Lightness), a* (redness), and b* (yellowness) system. The colorimeter was calibrated using a white calibration cap (CM‐A177, Konica Minolta Inc., Chiyoda, Tokyo, Japan) through a vacuum pouch the same as the sample storage vacuum pouch. Measurement of the white calibration cap was automatically completed five times with a preinstalled program on the spectrophotometer. The measurement and calibration were conducted in a temperature‐controlled laboratory space close to the optimal measuring temperature at 23°C. Four random locations on three randomly selected frankfurter cuts (20 mm thickness) were evaluated on Days 0, 15, 30, and 45.

### 2.5 Cure Color Ratio

The cured meat ratio (King et al. 2023) was determined by the percentage reflectance at 650 nm divided by the percentage reflectance at 570 nm, with greater ratios indicating more nitrosylhemochrome (cured meat pigment). The cure color ratio was measured using a spectrophotometer set to visible reflectance measurement (Shimadzu UV2600 UV–Vis Spectrophotometer coupled with UPC‐2600 multipurpose large sample component, Shimadzu Inc., Nakagyō‐ku, Kyoto, Japan). The spectrophotometer was calibrated with a standard white plate compatible with the Shimadzu UPC‐2600 through a vacuum pouch identical to the one used to store meat samples. Two slices of 20‐mm‐thick samples were stacked and stored in a vacuum pouch. The reflectance of the sample surface at 650 nm and 570 nm was measured triplicate and the average from each wavelength was calculated.

### 2.6 pH Measurement

pH measurements were conducted according to a method described for meat (Korkeala et al. 1986). Five grams of meat sample were blended with ultrapure water (resistivity of 18.2 MΩ.cm) at a 1:9 ratio using a polytron blender at 15,000 rpm. The mixture was then filtered through Whatman #1 filter paper and measured with a pH meter (Fisherbrand Accumet model 13‐620‐AE6; Fisher Scientific, Waltham, MA, USA). Calibration of the pH meter was performed using NIST‐certified potassium biophthalate buffer (pH = 4.0) and potassium monobasic and sodium hydroxide buffer (pH = 7.0).

### 2.7 Residual Nitrite and Nitrate Measurements

Residual NO_2_^−^ and NO_3_^−^ were analyzed using high‐performance liquid chromatography (HPLC) equipment (ENO‐20 NOx Analyzer, Eicom Inc., Kyoto, Japan) coupled with a temperature‐controlled autosampler (AS‐700, Amuza Inc., San Diego, C.A., USA) according to the method described by De González et al. (2012) with modifications. The HPLC analysis for NO_x_^−^ was designed based on the Griess nitrite test adopted by the AOAC. Absorption was measured at 540 nm by the UV–Vis detector preinstalled in the nitrite analyzer. Samples (processed meats and meat analogues) were powdered in liquid nitrogen and stored at −80°C until analysis. A 5‐g sample was weighed into 45 mL of pH 7.4 phosphate‐buffered saline (PBS) and then split into two equal volumes of slurries and centrifuged at 3500 × *g* at 4°C for 5 min (J6‐MI centrifuge equipped with JA‐25.50 rotor; Beckman Coulter, Indianapolis, IN, USA). After centrifugation, Supernatant (500 μL) from each slurry and 500 μL of 100% methanol were mixed, transferred to a 1.5‐mL snap cap centrifuge tube (Catalog number: 3453 Snap cap low retention microcentrifuge tubes, ThermoFisher Inc., Waltham, MA, USA), and vortexed for 10 s at 3000 rpm with a digital vortex mixer (cat. no. 0215370, Fisher Scientific Inc., Hanover Park, IL, USA). The samples were then centrifuged for 16 min at 15,000 × *g* at 4°C (Eppendorf 5424 centrifuge, Brinkmann Instruments, Westburg, NY, USA). Supernatants (200 μL) were pipetted into 96‐well plates for quantification with the HPLC equipment described above. Quantitative data (area under the curve) were analyzed with PowerChrom (version 16.0, New South Wales, Australia). HPLC carrier pump speed was set at 40 mL/h, and reactor pump speed was set at 13.2 mL/h. A calibration curve was created using 2, 4, 8, and 16 ppm of HPLC‐grade sodium nitrite and sodium nitrate. A sodium nitrite standard (8 ppm) was tested at the start and end of each run.

### 2.8 Total and Cured Meat Pigments Measurement

Total pigments, cured pigments, and cure efficacy measurements and calculations were conducted according to the meat pigments measurement guidelines, with modifications based on the AMSA Color Measurement guidelines (King et al. 2023). A 200‐g meat sample (cooked frankfurter) was minced using a commercial mixer (Robot Coupe BLIXER2 Blixer Vertical Commercial Blender with a 2.5‐quart stainless steel bowl, Robot Coupe Inc., Vincennes, France) for 10 s. For nitrosoheme determination, 10 g of the minced sample were weighed into a 100 mL beaker containing 43 mL of a solution comprising 40 mL acetone and 3 mL MilliQ water. After intermittent mixing for 5 min, the sample was filtered through Whatman #3 filter paper (Cytiva Inc., Marlborough, MA, USA) into a 50 mL polyethylene tube. A 1.5 mL aliquot of the filtrate was transferred into a 1‐cm quartz cuvette for measuring absorbance at 540 nm. Nitrosoheme content, expressed as NO‐hematin, was calculated based on the formulation: sample A_540_ × 290. All measurements were conducted in duplicate.

For total heme pigment determination, 10 g of the minced sample was weighed into a 100 mL beaker with acidified acetone (40 mL acetone, 2 mL MilliQ water, and 1 mL of 37% hydrochloric acid). The mixture was stored at room temperature for 1 h with intermittent stirring, then filtered into a 1 cm quartz cuvette using Whatman #3 filter paper. Optical density was measured at 640 nm to determine total heme content, based on the formulation: sample A_640_ × 680. All measurements were duplicated. All steps were conducted in a laboratory with LED lighting that does not emit UV‐A/B (UV A/B intensity was measured at 0 mW/cm^2^). Curing efficacy was calculated as the percentage of nitrosohemochrome (expressed as ppm acid hematin) divided by total pigments (expressed as ppm acid hematin), multiplied by 100.

### 2.9 Consumer Sensory Panel

Three untrained consumer sensory panels, conducted over three consecutive days at the University of Wisconsin‐Madison Meat Science and Animal Biologics (MSABD), were approved by the University of Wisconsin‐Madison Institutional Review Boards (IRB approval 2022–1342). These panels took place between Days 14 and 21 postmanufacture to simulate the quality attributes of processed meats as presented to consumers in the commercial supply chain. Voluntary participants included university faculty, staff, students, and members of the general public, aged 18 and over, recruited through mass email distribution (approximately 87,000 invitations per study) and an IRB‐approved poster (IRB approval # 2022–1342).

Panelists were served samples in individual booths separated from the sample preparation area by a one‐way glass window. The light intensity in each booth, maintained between 1615 to 2153 lx to simulate ideal meat‐display lighting, was measured at the beginning and end of each full testing day using a portable industrial handheld color meter (Sekonic C‐7000 Spectrometer, Sekonic US, North White Plains, NY, USA).

Each panelist was served four treatments randomly selected from the five TRTs. Four samples were used to establish a balance between providing enough data points for analysis and preventing sensory fatigue. The random selection process included all possible combinations, utilizing 10 base designs to ensure that each control and treatment was sampled equally by the panelists. Samples were served one at a time, with potable water provided to cleanse the palate between tastings. Frankfurter samples were cut into approximately 2.6 cm‐thick sections and served hot (56.2°C–61.2°C) in a 5.1‐cm white sample cup with a clear plastic lid. Consumers evaluated the frankfurters on a 9‐point hedonic liking scale (1 = extremely dislike, 9 = extremely like) for color, aroma, and overall liking. For non‐meat aftertaste, consumers used a 5‐point scale (1 = none, 5 = extremely strong). Purchase intent was measured using a 5‐point scale (1 = definitely will not buy, 5 = definitely will buy).

### 2.10 Volatile Compounds Analysis

Volatile compounds (VOCs) analysis was conducted according to a method described by Wettasinghe et al. (2001) with modifications on sample weight. Multiple studies have been conducted on extraction methods and confirmed that steam distillation generally extracts more VOCs than solid‐phase microextraction methods (Hong et al. 2018; Madruga et al. 2009; Watkins et al. 2012; Xie et al. 2016).

Samples (200 g) collected on Day 15 during the consumer sensory evaluation period were ground by a commercial meat blender (Robot Coupe BLIXER2 Blixer Vertical Commercial Blender with 2.5 Quarts stainless steel bowl, Robot Coupe Inc., Vincennes, France). Ground samples (10 g) were mixed with 10 g of sodium chloride in a 200 mL volumetric tube specifically designed to fit in a fast steam distillation system (SCP DigiPREP Distillation System, SCP Science, Baie‐d'urfe, Canada). The distillation was conducted at 60% strength for 300 s. Distillate (100 mL) was mixed with 150 mL of methylene chloride in a 500 mL separatory flask and mixed vigorously, and allowed to stay at ambient temperature (23°C) for 2 h. The methylene chloride layer was vaporized under vacuum using a rotary evaporator (Rotavapor, Buchi, Flawil, Switzerland) at 39.6°C. When the contents reached a volume of approximately 5 mL, they were carefully removed from the rotary evaporator and mixed with 5 g of sodium formate to remove any water in the solution. The concentrate was subsequently transferred into a dark glass vial and stored at −80°C until analysis.

A gas chromatography with tandem mass spectrometry (GC–MS/MS) system coupled with an autosampler (Shimadzu GCMS‐TQ 8040NX with AOC‐20 plus autosampler Shimadzu Inc., Nakagyo‐ku, Kyoto, Japan) supplied with helium gas with a smart switch was used. Separation of VOCs was conducted by a general purpose fused silica low polarity, crosslinked diphenyl dimethyl polysiloxane phase column (Shimadzu SH‐I‐5MS Capillary Column, 30 m x 0.25 mm x 0.25um, Shimadzu Inc., Nakagyo‐ku, Kyoto, Japan) for semivolatiles, phenols, amines, residual solvents, drugs of abuse, pesticides, and PCB congeners with an operation temperature range of −60°C to 330°C/350°C. Oven temperature was programmed from 45°C to 240°C at a rate of 5°C/min with initial and final hold times of 5 min and 10 min, respectively. The total running time was 60 min. For the mass spectrometry detector, the electron ionization energy was set at 70 eV. Mass range, electron multiplier voltage, and scan rate were set at m/z 33–330, 1500 v, and 20,000 μ/s, respectively. Ionization source temperature was maintained at 230°C (Li et al. 2023).

VOCs were identified by matching mass spectral data of sample compounds with an Electron Spray (EI) NIST database (NIST 23 Tandem Mass Spectral Libraries). The area under the curve (AUC) was integrated using the Savitzky–Golay method with width and sensitivity setting at 2.4 s. Each integrated area was compared with the EI database based on spectrum similarity and then manually analyzed based on fragmentation patterns. The results of each TRT were integrated for comparison using Python (Python version 3.12.7, The Python Software Foundation) on Spyder (The Scientific Python Development Environment, version 6.0.1, Spyder‐IDE.org) as an Integrated Development Environment (IDE).

### 2.11 Statistical Analysis

Physiochemical property data including cured meat pigments (CMP), total meat pigments (TMP), salt, and proximate analysis (moisture, fat, and protein) data were analyzed on day 0 with measurements done in duplicate. Objective color (Commission Interational de I'Eclairage [CIE] L* [Lightness], a* [redness], and b*[yellowness]), cured color ratio, residual nitrite (NO_2_^−^), and pH were assessed on a 15‐day interval from Day 0 to Day 45 with measurements conducted in triplicate. Since the TRTs were only manufactured once, for the physiochemical analysis data, descriptive statistical analysis was performed to determine means and a measure of variation associated with the repeated measures. One‐way analysis of variance (ANOVA) was performed to study data from sensory evaluations. Data from consumer sensory analysis on finished products were analyzed using R (R version 4.3.3; R Core Team 2024). Least square means were used when sample sizes are unequal, or values are missing. The Tukey and Dunnett multiple comparison tests were used if significant differences were detected. Pearson correlation and principal component analysis (PCA) were used to analyze sensory attributes and volatile compounds. PCA dimensionally reduced all variables into two principal components, PC1 and PC2, to describe data relationships based on eigenvalues (from parallel analysis). The accumulation of PC1 and PC2 (at least 70% or more for a good representation) was evaluated for the feasibility of PCA analysis. Correlation coefficient (“*r*”) indicates positive or negative relationships.

## 3 Results and Discussion

### 3.1 Proximate Composition and Physiochemical Analysis

Table 2 presents the proximate analysis of frankfurter cured with various nitrite sources on Day 0. The compositional profiles (moisture, protein, and fat) of frankfurter cured with either conventional or organic vegetable‐sourced nitrite were similar to those of the control made with sodium nitrite (SN). The finished frankfurter moisture protein ratio was between 3.79 and 3.94: 1, similar to the United States Department of Agriculture (USDA) Food database indicating an acceptable quality standard (U.S. Department of Agriculture 2019). Total fat percentage and added water in finished products for all TRTs were between 16.96% to 17.56% and −1.14% to 7.31%, respectively, that meet the USDA requirements for frankfurter's standard of identity (U.S. Department of Agriculture 1990).

**TABLE 2:** Means of proximate analysis, total meat pigments, cured efficiency, and pH of frankfurters.

Treatment	SN	CEL	OCEL	SW	OSW
Moisture (%)	66.56 ± 0.39	67.39 ± 0.43	67.20 ± 0.32	66.79 ± 0.33	65.34 ± 0.12
Protein (%)	15.35 ± 0.20	15.02 ± 0.21	16.19 ± 0.21	16.96 ± 0.23	16.62 ± 0.23
Fat (%)	17.56 ± 0.24	17.19 ± 0.27	17.28 ± 0.23	16.96 ± 0.13	16.98 ± 0.23
Moisture protein ratio	3.79 ± 0.04	3.92 ± 0.05	3.89 ± 0.04	3.94 ± 0.04	3.94 ± 0.03
Cured meat pigments^a^	51.16 ± 0.38	44.83 ± 0.32	36.80 ± 0.54	46.81 ± 0.19	46.00 ± 0.00
Total meat pigments^a^	67.39 ± 0.15	63.376 ± 1.06	63.31 ± 0.66	62.08 ± 0.47	63.72 ± 0.01
Cured efficiency (%)	75.91 ± 0.03	70.74 ± 0.09	58.13 ± 0.00	75.39 ± 0.08	72.17 ± 0.00
pH	6.29 ± 0.01	6.27 ± 0.00	6.28 ± 0.01	6.27 ± 0.01	6.27 ± 0.0

*Note:* Standard errors (SEM) are displayed following “±” for each cell.

Abbreviations: CEL, celery; OCEL, organic celery juice; OSW organic Swiss chard; SN, sodium nitrite; SW, Swiss chard.

^a^ Expressed as ppm acid hematin.

Cured meat pigments were determined to range from 36.82 to 51.16 ppm acid hematin equivalent, while total meat pigments ranged from 62.08 to 67.39 ppm acid hematin equivalent, respectively, for SN, CEL, OCEL, SW, and OSW (Table 1). An acceptable conversion rate for heme pigments to the nitrosylheme form during curing is generally considered to be less than 80% (Pearson and Tauber 1984). The cure efficiency in treatments (TRTs) ranged from 58.13% to 75.91%. The Overall cure efficiency in TRTs was in alignment and slightly higher than the value reported in previous studies (Posthuma et al. 2018) possibly due to the usage of a cure accelerator and the relevant fresh age of the product (Sullivan et al. 2012).

### 3.2 Residual Nitrite and Objective Color

**FIGURE 1:**
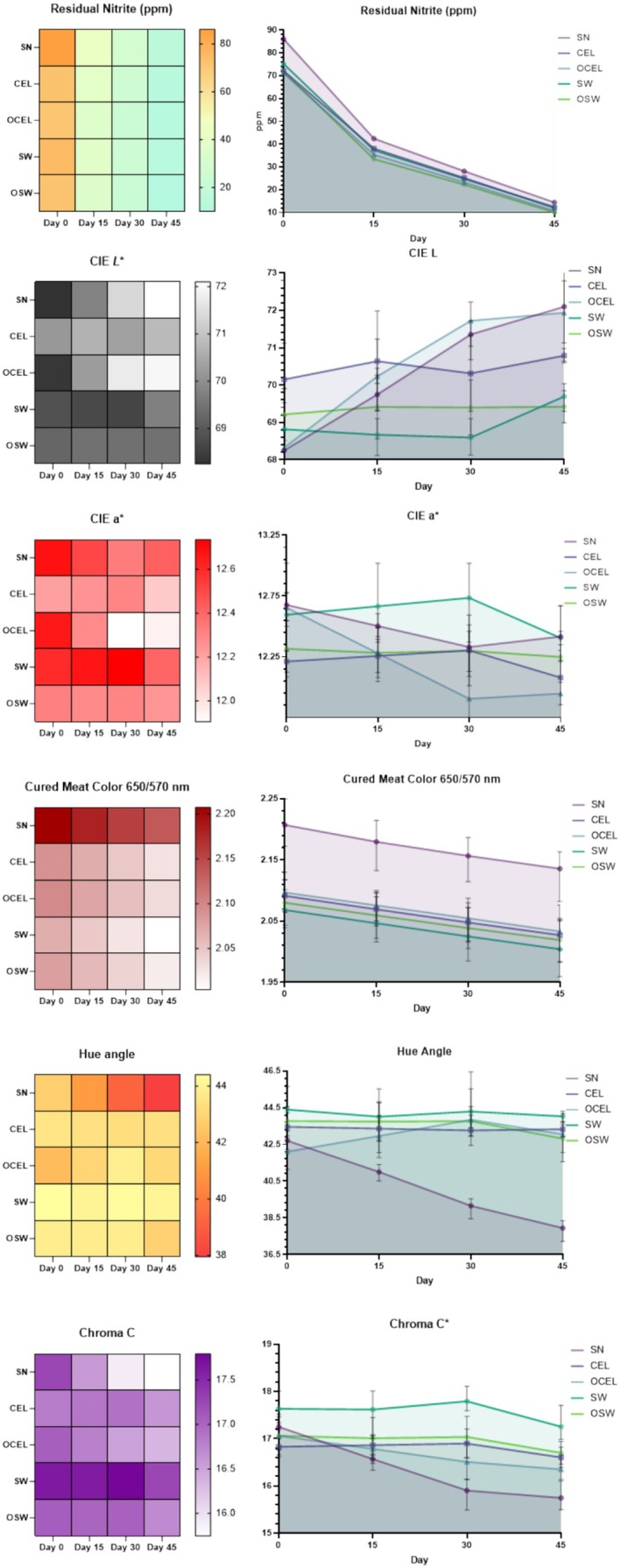
Means for the main effects of nitrite source (CEL, celery powder; OCEL, organic celery juice; OSW, organic Swiss chard powder; SN, sodium nitrite; SW, Swiss chard powder) on residual nitrite (measured in ppm), *L*, *a*, b*, cured meat color ratio (measured at a visual wavelength of 650/570 nm), hue angle, and Chroma C in frankfurters. Commission Internationale de l'Eclairage (CIE) *L*, *a*, b* color space, where L* represents lightness on a scale from 0 (black) to 100 (white), a* represents the red‐green axis (positive values indicate redness, negative values indicate greenness), and b* represents the yellow‐blue axis (positive values indicate yellowness, negative values indicate blueness).

Residual nitrite (NO_2_^−^) in frankfurters plays a crucial role in both food quality and safety (Shakil et al. 2022). Residual nitrite is considered a reservoir that retains the color by continually replenishing the cured meat color lost from oxidation and photooxidation in processed meats (Mancini 2013). In the current study, the amount of NO_2_^−^ initially ranged from 71.9 to 86.1 ppm on Day 0, gradually decreased to a range of 10.0 to 14.4 ppm over time by Day 45 after manufacture (Figure 1). The residual nitrite levels among the organic and conventional nitrite sources appeared relatively similar at each storage time and were in a similar range to those reported in a recent national survey on NO_2_^−^ and NO_3_^−^ (Sheng, Silva, Tarté, et al. 2025). As a general observation, the residual amount of NO_2_^−^ in plant‐sourced nitrite‐cured frankfurters suggests it is lower than SN. Polyphenols and other active compounds associated with plant‐sourced nitrite ingredients could interact with nitrite during curing. Polyphenols in plant curing powders have the potential to reduce nitrite to nitric oxide. Additionally, some phenolic compounds can undergo nitrosation under certain conditions, further reducing the amount of NO_2_^−^ (Niu et al. 2021; Rocha et al. 2009).

Overall color quality indicated good stability and aligned with previous similar studies that had adopted similar formulations (Posthuma et al. 2018; Tahmouzi 2016). In a study conducted by Posthuma et al. (2018), frankfurters cured with sodium nitrite exhibited higher CIE a* values and lower CIE b* values compared to those formulated with celery juice powder containing the same level of nitrite. Sodium nitrite leads to a more intense red color and reduced yellowness (Posthuma et al. 2018).

Cured meat color ratio in SN was observed to be higher than the rest of the plant‐sourced nitrite‐cured TRTs throughout the 45‐day sampling period. The initial cured meat color of SN was over 2.2, which indicated an excellent cure color (King et al. 2023) followed by OCEL (2.10), CEL (2.09), OSW (2.08), and SW (2.07). CIE a* value is another indicator of cured meat color that indicates similar trends in cured meat color ratio.

The hue angle signifies the color shift from redness (lower degree angle) to yellowness (higher degree angle) (King et al. 2023). Interestingly, in contrast to the plant‐based TRTs, the SN treatment shifted to a lower degree hue angle compared with the mean of alternative cured treatments (TRTs) which could be attributed to the loss of CIE b* during the sampling period. This color shift was further indicated by the Chroma C*, which employs CIE a and b* values to evaluate the color saturation. Among vegetable source nitrite TRTs, OSW exhibited better color saturation than the remaining TRTs during the sampling period; the difference was attributed to the overall higher CIE a* and b* values. OSW may contain more plant pigments than the conventional varieties. In a study on growing practices and nutritional quality, it was found that the chlorophyll content of conventionally produced chard (2.99 g/kg wet weight) was lower than that of organically produced chard (3.21 g/kg wet weight). The observed variation in the color impact of these curing agents on alternative cured frankfurters can be attributed to differences in chlorophyll content (Ivanović et al. 2019). Additionally, carotenoids, another category of plant pigments, contribute yellow to orange hues and may influence the color attributes of cured meats. Several studies (Dhakal et al. 2021; Indu et al. 2024; Søltoft et al. 2011) have demonstrated that organic farming practices, which forego pesticides and utilize different fertilizers, can enhance the content of secondary metabolites such as carotenoids. The variations of plant pigments could explain the intricate relationship between growing practice and the resulting color properties of cured meat in this study.

### 3.3 Sensory Evaluation

A total of 114 panelists were recruited to attend one of the sensory evaluation sessions held at the MSABD sensory laboratory. The age distribution of the participants was as follows: 16% were aged 18–24, 30% were aged 25–34, 10% were aged 35–44, 19% were aged 45–54, 11% were aged 55–64, and 14% were over 64. Regarding frankfurter consumption, 6% of the consumers indicated they consumed frankfurters at least once per week, 25% indicated several times per month, 29% indicated once per month, and 40% indicated less than once a month.

**FIGURE 2:**
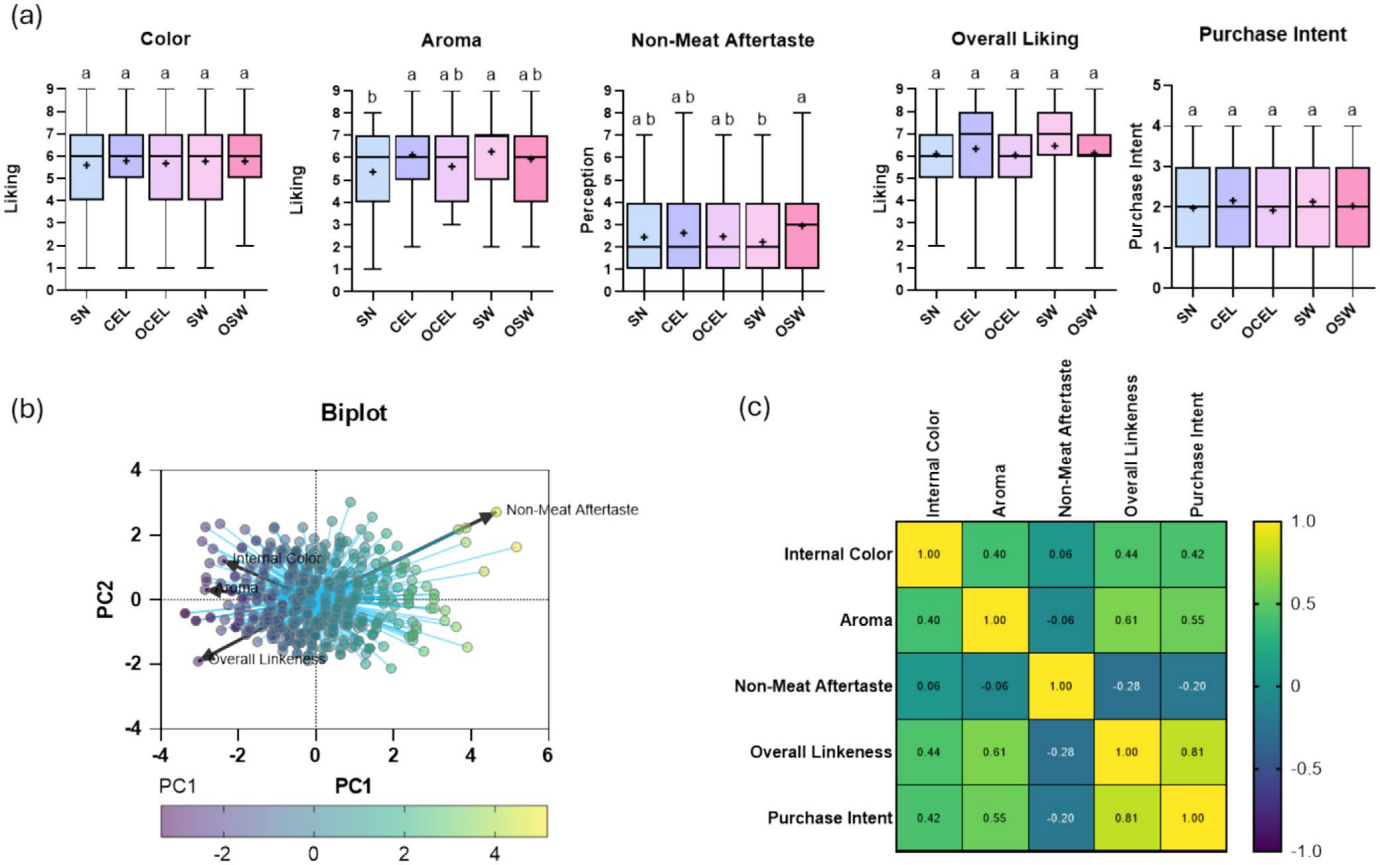
Sensory attributes analysis of the control (C) and all treatments (TRTs) of frankfurters. (a) Boxplot of sensory attributes of the control (C) and all treatments (TRTs). (b) Biplot of frankfurter sensory attributes using PCA analysis. (c) Correlation analysis of all sensory attributes of frankfurter. In (c), different letters denote a significant difference (*p* < 0.05), and “^+^” indicate the mean value. Consumer sensory 9‐point hedonic liking scales: 1 = extremely dislike to 9 = extremely like for color, aroma, and overall liking. Non‐meat aftertaste scale: 1 = none to 9 = Extremely Strong. Purchase intent scale: 1 = Definitely will not buy to 5 = definitely will buy.

Sensory evaluation revealed no differences (*p* > 0.05) for color, overall liking, and purchase intent between traditional sodium nitrite‐cured frankfurters and alternative cured frankfurters, whether from organic or conventional vegetable curing powders (Figure 2a). These findings were aligned with previous studies that demonstrated that the addition of plant source curing powder had no adverse effects on the sensory attributes of cured meats using celery powder (Jin et al. 2018), cherry and lime powder (Xi et al. 2012), *Ocimum rarissima* leaf extract (Akwetey et al. 2021), and grape seeds extract (Parrini et al. 2019) at proper concentrations. Aroma liking was greater (*p* < 0.05) for CEL and SW than SN (Figure 2a). In terms of non‐meat aftertaste, OSW exhibited a higher perception (*p* < 0.05) than SW. Interestingly, the OCEL did not exhibit a higher perception (*p* < 0.05) than CEL, very likely due to organic celery powder potentially being deodorized and not containing celery‐like aromatic compounds, according to a recent study on volatile compounds in plant‐based meat curing ingredients (Sheng, Silva, Ricke, et al. 2025).

PCA analysis reduces dimensionality and identifies underlying patterns of relationship between internal color, aroma, non‐meat aftertaste, and purchase intent. PCA analysis (Figure 2b) indicated that higher internal color and aroma scores were positively associated with overall liking and purchase intent of the products. Additionally, higher perceptions of non‐meat aftertaste were strongly positively associated with overall likability and purchase intent. This finding aligns with previous studies that formulated processed meats with plant‐sourced nitrites, which reported undesirable aftertastes in turkey bologna (Djeri and Williams 2014), reduced sodium boneless ham (Pietrasik et al. 2016), and regular boneless ham (Sindelar et al. 2007).

To examine the correlation between these attributes more closely, a Pearson correlation study was conducted (Figure 2c) to study the linear relationship between two variables. The analysis revealed that non‐meat aftertaste was negatively associated with overall liking (*r* = −0.28) and purchase intent (*r* = −0.20). In contrast, internal color was positively associated with overall liking (*r* = 0.44) and purchase intent (*r* = 0.42). The most important factor influencing consumer overall liking and purchase intent for frankfurters was aroma (*r* = 0.61 and *r* = 0.55, respectively). A previous study on frankfurters asserts that an increased level of vegetable juice added was associated with decreased consumer liking of frankfurters (Sindelar et al. 2007). In this study, we found that the organic‐produced plant source curing powder (OSW) may lower consumer liking of the product compared to the conventional produced plant source curing powder (SW) when formulated at the same nitrite concentration. To further understand the underlying reasons, we conducted a volatile compounds (VOCs) analysis on the TRTs of frankfurters.

### 3.4 Volatile Compounds Analysis

**FIGURE 3:**
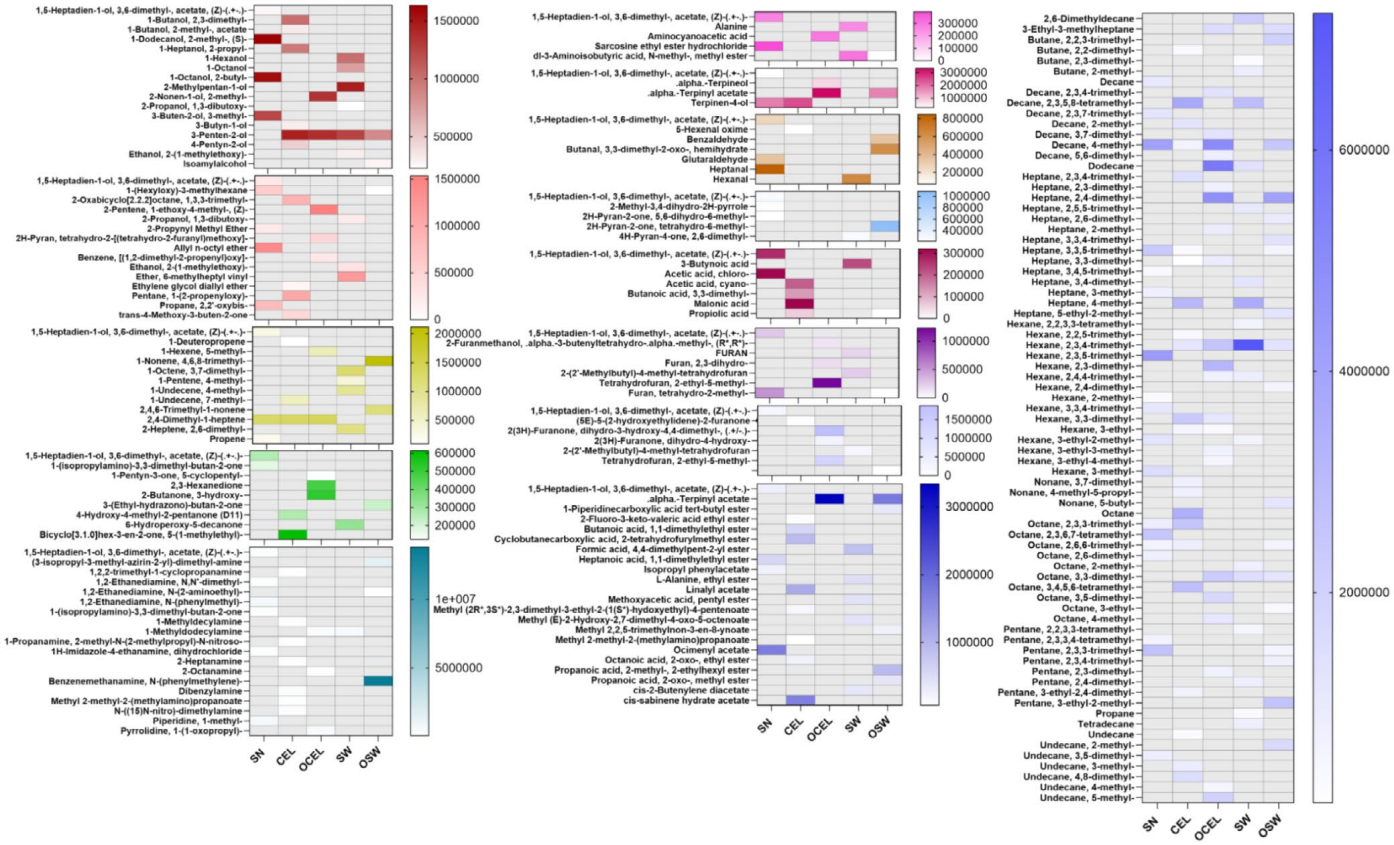
Comparative analysis of volatile compounds (VOCs) in frankfurters treatments. CEL, celery; OCEL, organic celery juice; OSW, organic Swiss chard; SN, sodium nitrite; SW, Swiss chard.

A total of 1203 VOCs were identified, of which 271 compounds exhibited a spectrum fragmentation pattern with 80% similarity to compounds in the current National Institute of Standards and Technology (NIST) electron ionization (EI) database. A distinct aromatic compound profile was established from identifiable VOCs among all TRTs (Figure 3).

**FIGURE 4:**
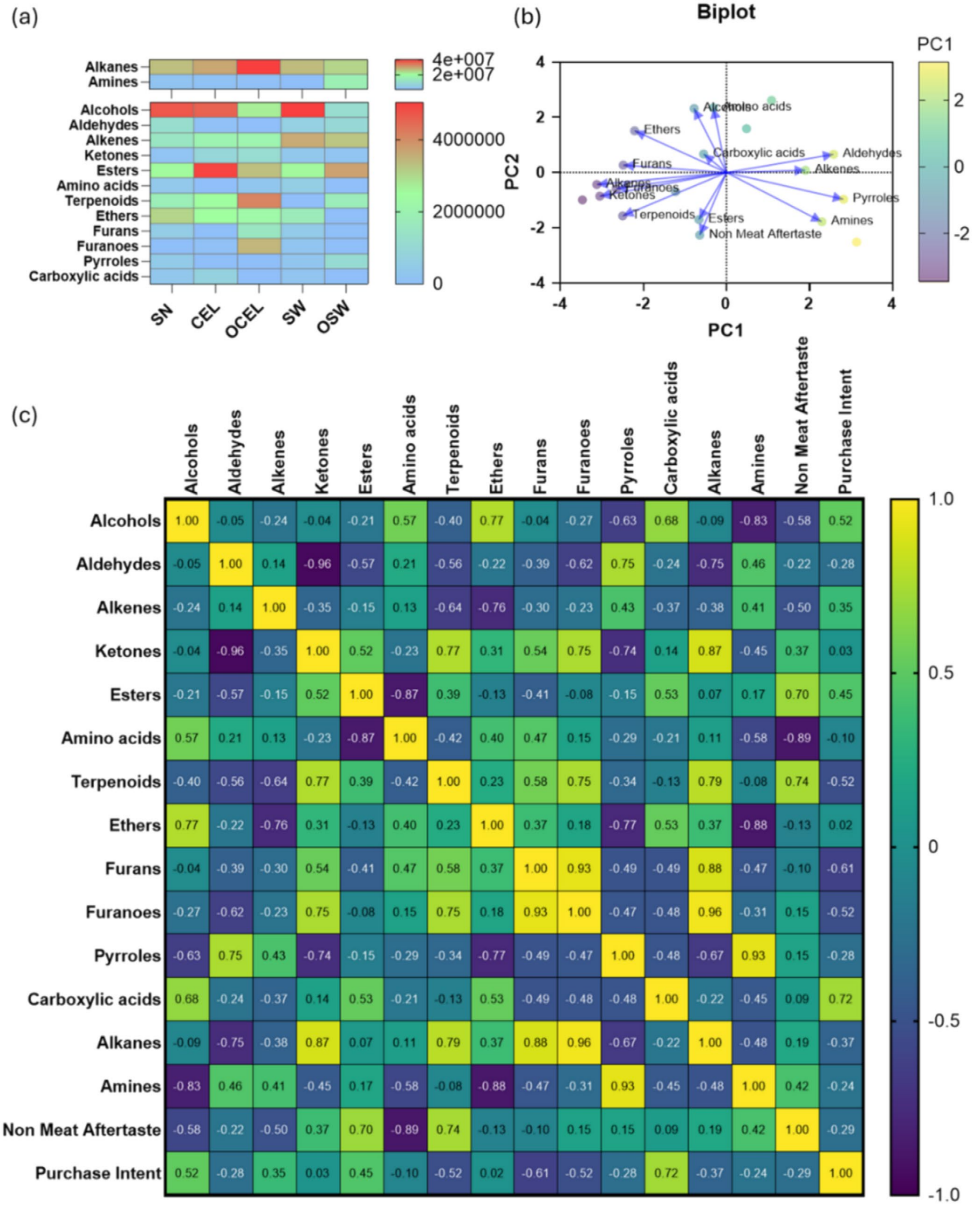
Volatile compounds in frankfurters that were cured with different sources of nitrite. (a) Relative concentrations of key volatile compounds in control and all treatments of frankfurters. (b) Biplot of frankfurter VOCs and sensory attributes (non‐meat aftertaste and purchase intent). (c) Correlation analysis of frankfurter VOCs and negative sensory attributes.

Alkanes and amines were identified to be the most abundant compounds in frankfurters, followed by alcohols, aldehydes, alkenes, ketones, esters, amino acids, terpenoids, ethers, furans, furanones, pyrroles, and carboxylic acids (Figure 4a). A biplot from PCA analysis (first two components consist of 76.56% of total variance) in Figure 4b indicated that esters and terpenoids were strongly positively correlated with non‐meat aftertaste, whereas alcohols, amino acids, and aldehydes were strongly negatively correlated with non‐meat aftertaste.

A detailed correlation study using Pearson's *r* correlation analysis as laid out in Figure 4c indicates similar findings with terpenoids positively correlated with nonmeat after taste (*r* = 0.74) and negatively correlated with purchase intent (*r* = −0.10). The presence of α‐terpineol (3‐penten‐2‐ol) and α‐terpinyl acetate in the alternatively cured treatments likely contributed to this observation. Notably, α‐terpineol was identified in all alternative treatments but was absent in the sodium nitrite (SN) cured samples (Figure 4). α‐Terpineol is a natural tertiary monoterpenoid alcohol widely used in the flavors and fragrances industry due to its unique sensory attributes (Sales et al. 2020), characterized by a pleasant odor reminiscent of lilacs (Khaleel et al. 2018). However, some pleasant fragrances may not contribute to a desirable aroma for meat consumers. A cognition‐based framework study indicated that consumer's perception of odor is associated with individuals' episodic memories and past experiences (Morrin and Ratneshwar 2000). Familiarity is one of the basic dimensions through which individuals perceive smells (Rabin and Cain 1984). The terpenoid notes (typically herbal, woody), which are not typically associated with the flavor of processed meat, may explain the perception of non‐meat aftertaste in some TRTs (Šojić et al. 2023). This non‐meat aftertaste was negatively associated with overall liking and purchase intent.

Esters were another group of chemicals that were strongly positively associated (*r* = 0.70) with non‐meat aftertaste while increased purchase intent (*r* = 0.45, Figure 4). Esters are formed through the esterification of carboxylic acids and alcohols during thermal processing of meat products and are considered products of lipid degradation (Mottram 1998). Gardner and Legako (2018) conducted a study on volatile compounds in beef and found that certain esters contribute to the desirable flavors in cooked meat, leading to higher consumer preference and purchase intent. Esters were not identified in OSW, but were distributed differently in other TRTs. 3‐methyl esters, which contributed to a fruity flavor, were found in the conventional source of chard that is known to be correlated with beef flavor (Mallick et al. 2021).

Alcohols were one of the most abundant chemicals identified in this study among TRTs (Figure 3). Alcohols were negatively correlated with non‐meat aftertaste (*r* = −0.58), but moderately positively correlated with purchase intent (*r* = 0.52). Moreover, alcohols were more abundant in the conventional curing powder than in the organic‐produced varieties (Figure 4a). Linear alcohols in meat develop under heating and oxidation, and branched‐chain alcohols are derived from the decomposition of branched aldehydes (Xie et al. 2022). Alcohols derived from unsaturated fatty acids, such as hexanol and 1‐octanol, have very low detection thresholds and may significantly influence the flavor of meat (Iglesias et al. 2009). These alcohols can enhance meat flavor during reheating. In a study on cooked beef, Mallick et al. (2021) concluded that alcohols develop within the internal structures of cooked meat and may further convert into other volatile compounds during reheating.

Furans and furanones exhibited a weak association with non‐meat aftertaste (*r* = −0.10 and 0.15, respectively) but were moderately negatively associated with purchase intent (*r* = −0.61 and −0.52, respectively) (Figure 4c). Furans and 2‐ethyl‐5‐5methyl tetrahydrofuran were presented in OCEL and SW cured frankfurters at different concentrations. Furans can be formed in meat through thermal degradation of carbohydrates, oxidation of carboxylic acids and polyunsaturated fatty acids, and Maillard reactions (Farmer et al. 1999). Tetrahydro‐2‐methyl furan was presented in SN cured traditional frankfurter. In cooked meats, furans and furanones contribute to the characteristic “meaty” and “fruity” aromas. From a chemical structure perspective, the presence of sulfur atoms in furans can enhance the “meaty” aroma, and a cyclic structure of furans may feature the “fruity” aroma (Wailzer et al. 2016).

## 4 Conclusions

Curing ingredients play a crucial role in the development of the color and sensory attributes of frankfurters. While the color differences in frankfurters cured with various ingredients were noticeable, they did not significantly influence overall consumer liking of the products. Organic and conventional crop‐growing practices impact consumer perceptions of aroma and non‐meat aftertaste. These curing ingredients may contribute to perceptible differences and the development of distinct volatile organic compounds (VOCs) profiles. Processing and manufacturing alternative curing ingredients may reduce or eliminate plant volatile compounds that reduce the non‐meat aftertaste perception of finished organic cured meat products. However, consumers exhibited limited differences and preferences regarding purchase decisions based on these attributes.

## Author Contributions

**Siyuan Sheng:** data curation (lead), formal analysis (lead), investigation (lead), methodology (lead), visualization (lead), writing – original draft (lead). **Steven C. Ricke:** resources (equal), writing – review and editing (equal). **Erin M. Silva:** funding acquisition (lead), project administration (equal), resources (equal), writing – review and editing (equal). **James R. Claus:** project administration (equal), resources (equal), writing – review and editing (equal).

## Conflicts of Interest

The authors declare no conflicts of interest.

## Data Availability

Data will be made available on request from corresponding authors.
